# Simple and Environmentally Friendly Fabrication of Superhydrophobic Alkyl Ketene Dimer Coated MALDI Concentration Plates

**DOI:** 10.1007/s13361-017-1657-4

**Published:** 2017-04-12

**Authors:** Joakim Romson, Johan Jacksén, Åsa Emmer

**Affiliations:** 1 KTH Royal Institute of Technology, School of Chemical Science and Engineering, Department of Chemistry, Analytical Chemistry, Stockholm, Sweden; 2 KTH Royal Institute of Technology, School of Chemical Science and Engineering, Department of Chemistry, Organic Chemistry, Stockholm, Sweden; 3 Biotage Sweden AB, Uppsala, Sweden

**Keywords:** MALDI, Concentration plates, Alkyl ketene dimer

## Abstract

Here we present a method to manufacture peptide-concentrating MALDI-plates with alkyl ketene dimer (AKD) as a new superhydrophobic coating. The fabrication of the hydrophobic plates included application of AKD by airbrush, and negative contact printing to generate the concentration sites. Deposited sample droplets were contained within the prestructured sites, and self-adjusted onto the site if slightly misplaced. No AKD contamination was observed, and the plates could easily be cleaned and regenerated. The S/N values for four model peptides was about twice as high compared with a standard steel plate and a commercial concentration plate.

**Electronic supplementary material:**

The online version of this article (doi:10.1007/s13361-017-1657-4) contains supplementary material, which is available to authorized users.

**Graphical Abstract:**
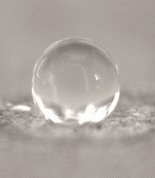
ᅟ

## Introduction

It is universally recognized that bioanalysis is dependent on sensitive and selective methods due to the often limited access of sample, and low concentrations of the analytes in complex matrices. Thus, mass spectrometry (MS), especially electrospray ionization (ESI) and matrix assisted laser desorption/ionization (MALDI) MS are cornerstones for e.g., cellular research, proteomic studies, clinical purposes, and screening for potential disease markers. Sensitivity with MALDI is affected by sample preparation, but even after enrichment the signals can be below the detection limit.

Improved target design is an attractive way to increase sensitivity through, e.g., enhanced homogeneity and concentration [1, 2]. Specially fabricated targets [1–4] have been used for sample preparation and enrichment. A convenient alternative is coated targets relying on hydrophobic/hydrophilic phenomena, e.g., [5–9], including commercially available plates. Nonetheless, commercial plates are expensive, restricted in the spot configuration/dimensions, and limited in lifetime. Thus, options offering simple, cheap, and customizable manufacturing of plates are desirable. Here, we present an alkyl ketene dimer (AKD) structured target that is easily fabricated. AKD is chosen because of its low toxicity, eco-friendliness, low cost, and accessibility (used extensively for paper sizing) [10]. Other prerequisites were the easy regeneration and reuse, and use of green solvents.

## Experimental

### Chemicals and Materials

[Glu1]-fibrinopeptide B (GFpB, 1570.68 Da), angiotensin I, (AngI, 1296.68 Da), angiotensin II, (AngII, 1046.54 Da), neurotensin (NT, 1672.92 Da), and trifluoroacetic acid (TFA) were from Sigma-Aldrich, Stockholm, Sweden. Alkyl ketene dimer (AKD, Online Resource 1) was from Eka Chemicals (Bohus, Sweden) and was a gift from Cellutech AB, Stockholm, Sweden [11]. MALDI plates [ground steel (GS) and AnchorChip (AC)], 2,5-dihydroxy benzoic acid (DHB), and α- cyano-4-hydroxy-cinnamic acid (HCCA) were from Bruker Daltonics (Bremen, Germany). Water was from a Millipore Synergy 185 system, (Millipore Corp., Bedford, MA, USA).

### AKD Concentration Plate Fabrication

Ground steel MALDI plates (8.1 cm × 12.3 cm) were step-wise coated with AKD; the plates were preheated with a heat gun (to avoid AKD precipitation), manually submerged vertically in a stirred beaker with 30 mg/mL AKD in ethanol (1 L, ca. 78 °C, 10 min), and vertically air-dried until cool (ca. 10 min). Care was taken to minimize solvent evaporation; any ignition sources must be avoided. This created a thin and smooth hydrophobic AKD layer on the surface. For surface roughness and increased hydrophobicity [12], additional AKD solution was applied with a heated airbrush from ca. 15 cm distance. Five airbrush layers were applied, each allowed to dry ca. 0.5 min.

A 10-μL plastic pipette tip, melted at the tip to form a spherical shape, was used as printing substrate (Online Resource 2). By pressing the tip against the plate, the topography was removed and the AKD layer thinned, yielding concentration sites with a less hydrophobic surface, and diameters depending on substrate size and printing pressure. Printing was performed with either of two positioning tables: (1) three M-ILS50CC stages with an ESP301 Motion Controller, Newport (Darmstadt, Germany); (2) TIXY 200 XY-platform (Newport), with MM53M5EX extended Motorized MicroMiniTM Stage, National Aperture (Cambridge, UK), and Arduino DUE (Budapest, Hungary).

To clean and regenerate the AKD plates for reuse, the coating was stripped by wiping and rinsing with ethanol, 20 min sonication in ethanol, and a second rinse. The plates were additionally cleaned according to a standard protocol (Bruker Daltonics MALDI preparation protocols – Life Science, ver. 100623).

### Evaluation of Plate Performance

The hydrophobicity of the AKD plate was evaluated by digitally measuring [software: ImageJ, Wayne Rasband, National Institute of Mental Health (Bethesda, MD, USA)] the contact angle of 10 × 1 μl water droplets. The droplets were applied with pipette and photographed [LU Plan Fluor 5x/0.15 A lens (Nikon, Japan), CM-10 L-IM camera (Nikon, Japan), XC-ES30CE CCD-sensor (Sony, Japan)].

The AKD plate was compared with a standard commercial GS plate, and a commercial AC plate. Three model peptide samples were prepared: 1.0 nM (for calibration), 0.1 nM, and 0.05 nM each of AngII, AngI, GFpB, and NT. The samples were mixed 1:1 with matrix (DHB 20 mg/mL, HCCA saturated, 0.7 mg/mL (Bruker Daltonics protocols), and 0.35 mg/mL, in 30:70 (v/v) acetonitrile:TFA 0.1% in water, that is TA30). A lower DHB concentration (5 mg/mL in water) than suggested in the protocols was tested on a smaller data set (n = 4) with automated acquisition. HCCA recrystallization was performed with 6:3:1 (v/v) ethanol:acetone:TFA 0.1%. A pressurized etched capillary [150/50 μm (o.d./i.d.), CM Scientific (Silsden, UK)], and a positioning table were used for sample deposition (0.5 μL). For consistency, the sample preparation followed the standard protocol for GS plates. Protein LoBind Eppendorf tubes [Eppendorf (Horsholm, Denmark)] were used, and capillaries were pre-equilibrated with the solutions to avoid adsorption. Concentration sites with 400 μm diameter were fabricated on the AKD plates to mimic the AC plate 400 μm anchors. Signals were acquired manually and automatically in reflector mode with detection between *m/z* 1000 and 2000 (Bruker ultrafleXtreme, flexControl, Bruker Daltonics).

For manual acquisition, calibration was performed and laser strength manually optimized. Spectra were acquired from 10 previously unused spots with partial sample random walk, (5 × 100 shots, 20 positions). The positions were chosen during acquisition for highest S/N.

An automated analysis sequence was also used where calibration was performed and laser strength was optimized (10 unused spots, 100 shots at 100 positions). The positions were chosen by random walk within a 500 μm radius from the center. Data was processed in flexAnalysis (Bruker Daltonics) with a S/N threshold of 2. Statistical analysis (Microsoft Excel mac 2011 with AnalystSoft Inc. StatPlus mac V5, free version) and average S/N for the peptide signals were used to quantify performance. The two-tailed t-test (t evaluated at P = 0.05 and 0.1), with the null-hypothesis that the mean S/N values do not differ significantly between the plates, was used. Normal distribution was assumed; an F-test was performed to check variances, and outliers were not excluded.

## Results and Discussion

### Characterization of the AKD Plates

**Figure 1:**
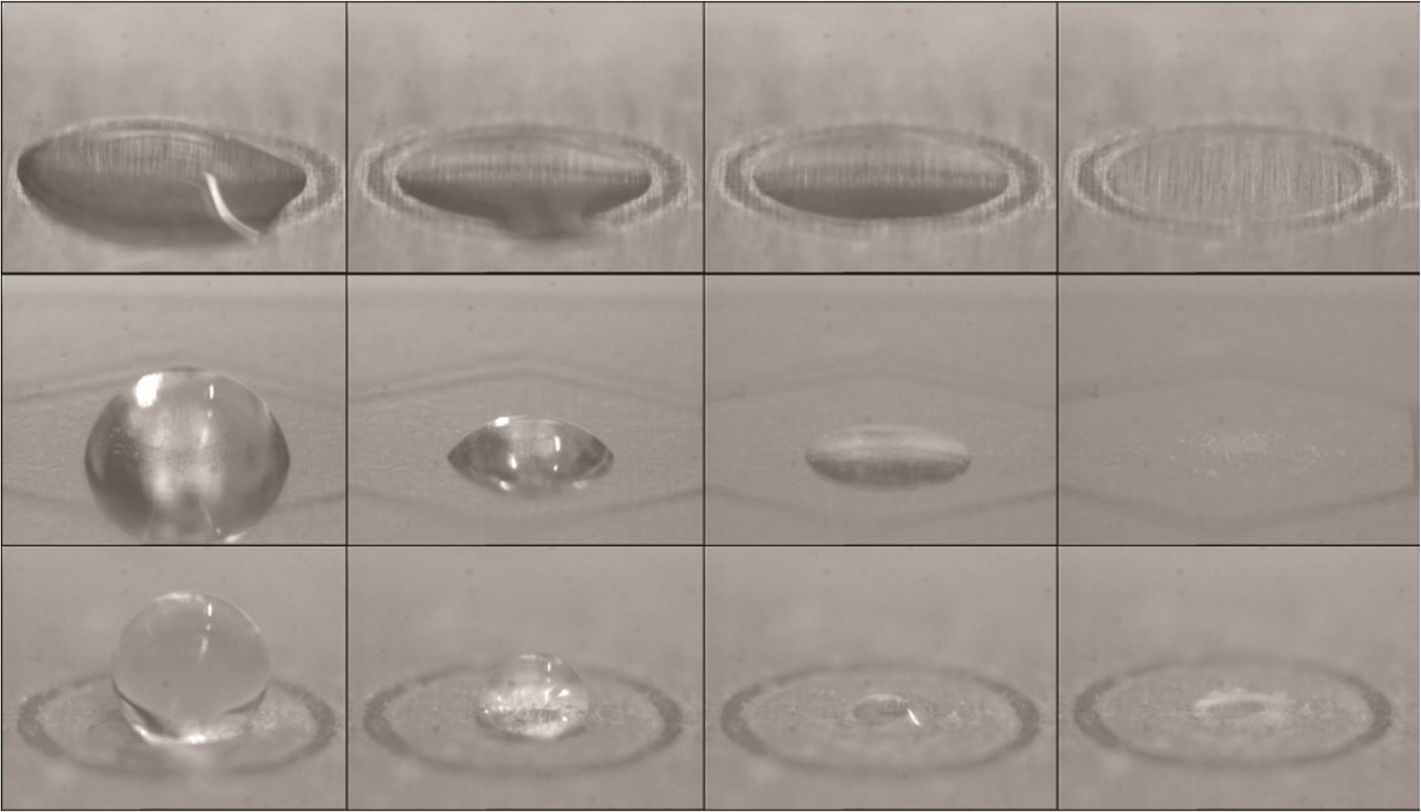
Photographs of drying 0.5 μL sample droplets (100 nM of each peptide and 0.35 mg/mL HCCA) on the three MALDI plates. GS plate at the top, AC in the middle, and AKD at the bottom

Initial tests showed that superhydrophobicity (≥150° contact angle [13]) could be obtained (by application of 25 or more airbrush layers), but while the increased roughness yielded higher contact angles, the surface obtained by so many air-brushed layers was uneven, causing peak broadening and lowered S/N. Five layers resulted in a somewhat lower hydrophobicity (average contact angle of 131.7°, SD = 5.305° (4%), n = 10), adequate concentration effect, and low peak broadening. As the droplets were positioned in surface depressions, the true contact angle might be even higher than measured. Evaporating sample droplets on GS-, AC-, and AKD plates are shown in Figure 1 (photographs of the sites with and without droplets could be seen in Online Resource 3).

The sites on the AKD plate were slightly oval due to the printing substrate shape. The longest diameter was measured. Aiming for 400 μm diameter, the average obtained was 411.9 μm, SD = 12.54 μm (3%), n = 10. The AC plate had an average diameter of 415 μm, SD = 17.48 μm (4%), n = 10, for the nominally 400 μm anchors. While AC plate anchors expose steel, AKD plate sites are created by the removed topography and thinner AKD layer. Although this will not create a completely hydrophilic anchor, as in AC plates, the change is sufficient to accomplish a considerable decrease of the contact angle. The droplets self-aligned to the AKD plate sites at quite large positioning errors during deposition, and the droplets retracted onto the sites during drying.

### Manual Spectral Acquisition

DHB 20 mg/mL TA30, HCCA saturated, HCCA 0.7 mg/mL, and HCCA 0.35 mg/mL TA30, were evaluated as matrices for manual spectral acquisition. HCCA saturated and 0.7 mg/mL gave higher S/N than DHB 20 mg/mL, and HCCA 0.35 mg/mL outperformed higher concentrations of matrix for all plates and sample concentrations. Around 50% laser power was optimal for all plates. No contamination from AKD could be observed for any matrix, even at high laser power. Further, no detrimental effects on the accuracy were noted.

**Figure 2:**
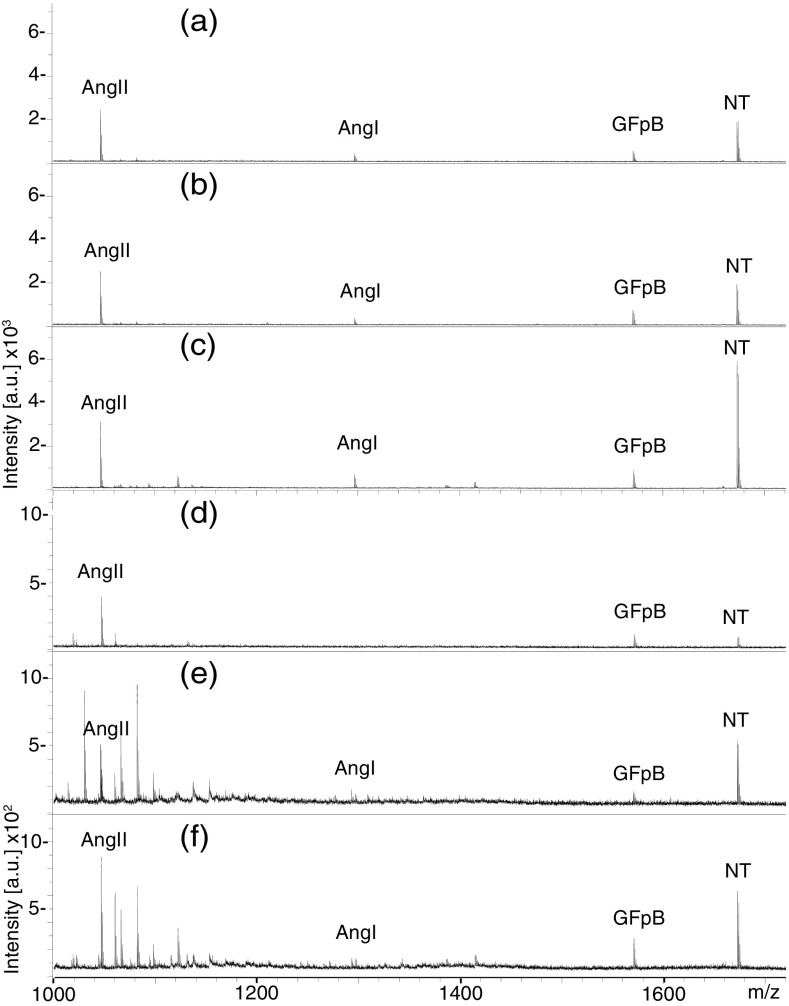
Spectra showing highest S/N values obtained using manual acquisition **(a)**–**(c)**, and using automated acquisition **(d–(f)**; 0.05 nM each of AngI, AngII, GFpB. and NT. (a and d) GS plate, (b and e) AC plate, (c and f) AKD plate

Sweet spots were seen on GS and on AKD, but with significant difficulty on AC. This probably partly explains why AC sometimes performed worse than GS, and the higher relative standard deviation for AC (Table 1) using manual spectral acquisition. Overall, AKD produced 1.54 times higher S/N values compared with GS, and 2.31 compared with AC, (Table 1, and Online Resource 4). The best spectra obtained at 0.05 nM peptide concentration are compared in Figure 2 (see Online Resource 5 for 0.1 nM). The sensitivity was high for all AKD plates evaluated (above S/N = 10) for all four peptides at 12.5 amol. Solvent evaporation during the slow (10 s) deposition could give a small error in the measured deposited volume (droplet diameter), but the relative sensitivities remain unchanged.

**Table 1:** Comparison of S/N Value Ratios for the Four Peptides Obtained Using the AKD Plates in Relation to the Two Other Plates (GS and AC). P is the Probability of Falsely Rejecting the Null Hypothesis

Ratio	0.1 nM				0.05 nM				Overall	
	AKD/GS	P %	AKD/AC	P %	AKD/GS	P %	AKD/AC	P %	AKD/GS	AKD/AC
Manual										
AngII	0.943	>10	1.61	< 5	1.07	> 10	1.54	< 5		
AngI	1.32	< 5	4.37	< 5	1.96	< 5	2.88	< 5		
GFpB	1.28	> 10	0.775	> 10	1.25	< 5	1.30	< 5		
NT	1.66	< 5	2.89	< 5	2.87	< 5	3.08	< 5		
Average ratio	1.30		2.41		1.78		2.20		1.54	2.31
SD	0.255		1.36		0.708		0.788			
RSD %	19.6		56.4		39.7		35.8			
Auto										
AngII	1.90	< 5	2.04	< 5	1.44	< 5	3.03	< 5		
AngI	2.53	< 5	1.23	< 10	1.30	> 10	1.18	> 10		
GFpB	2.38	< 5	1.47	> 10	1.71	< 5	2.65	< 5		
NT	3.46	< 5	1.42	> 10	3.09	< 5	2.04	< 5		
Average ratio	2.57		1.54		1.88		2.23		2.22	1.88
SD	0.564		0.304		0.711		0.698			
RSD %	22.0		19.7		37.7		31.4

### Automated Spectral Acquisition

For automated acquisition standard protocol for AC plate, HCCA 0.7 mg/mL TA30 gave the best results, and was used. While DHB at 20 mg/mL gave worse S/N, 5 mg/mL performed similarly to HCCA with automated aquisition. The AKD plate gave up to 50% higher S/N compared with AC using DHB 5 mg/mL (Online resource 6). HCCA recrystallisation did not increase S/N for any of the three plates.

The AKD plates gave the most consistent performance, and the lowest RSD using the automated procedure. This indicates homogeneity of the surface hydrophobicity, repeatability of the site printing, and sample deposition. Overall, AKD produced 2.22 times higher S/N values compared to GS, and 1.88 compared with AC (Table 1 and Online Resource 4). As expected, both AKD and AC showed better S/N than GS. This is also the case when the best spectra obtained are compared (see Figure 2 for 0.05 nM and Online Resource 5 for 0.1 nM).

It could be suspected that there is a risk for interaction of hydrophobic analytes with the AKD surface. Comparing the signals for the less hydrophilic Ang I and Ang II to signals for the more hydrophilic NT and GFpB obtained for the three different plates did not give any evidence for this (Figure 2 and Online Resource 5).

## Conclusions

A simple and repeatable fabrication method was developed to obtain AKD structured concentration MALDI plates with possibility of customized patterns. The hydrophobicity of the AKD plates could be optimized through the number of layers applied to improve S/N values. Furthermore, the fabrication of the AKD plate is environmentally benign, due to the solvent (ethanol), and the material. They are also economically viable since the coating is virtually indefinitely renewable. The AKD plate consistently showed significantly higher S/N compared with a nonstructured GS plate and a concentration AC plate in a comparison including four model peptides at two concentrations (0.05 nM and 0.1 nM). This result did not depend on using manual or an automated acquisition.

## Electronic supplementary material

Supporting Information available include AKD structure, photographs of the sites on the three plates, printing substrates images, and manufacturing details.

Online Resource 1 (PDF 628 kb)

Online Resource 2 (PDF 484 kb)

Online Resource 3 (PDF 589 kb)

Online Resource 4 (PDF 592 kb)

Online Resource 5 (PDF 633 kb)

Online Resource 6 (PDF 66 kb)
