# Supplementary material for: Simple and Environmentally Friendly Fabrication of Superhydrophobic Alkyl Ketene Dimer Coated MALDI Concentration Plates
Source: J Am Soc Mass Spectrom. 2017 Apr 12;28(8):1733–6. doi: 10.1007/s13361-017-1657-4 (PMC5507968; doi:10.1007/s13361-017-1657-4)

Online resource 1 – EMS_1

Journal of the American Society for Mass Spectrometry

“Simple and environmentally friendly fabrication of superhydrophobic alkyl ketene dimer coated MALDI concentration plates”

Joakim Romson, Johan Jacksén and Åsa Emmer*
*Corresponding author: aae@kth.se, KTH Royal Institute of Technology, School of Chemical Science and Engineering, Department of Chemistry, Analytical Chemistry, Stockholm, Sweden

EMS_1. Chemical structure of AKD. R_1 and R_2 are C_{16} or C_{18} alkane chains.

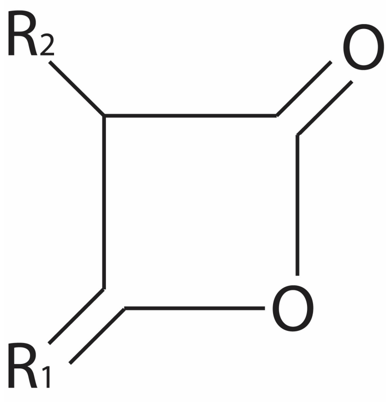

Supplement: Supplementary file 1 — (PDF 628 kb) [file 13361_2017_1657_MOESM1_ESM.pdf]
