# Supplementary material for: Simple and Environmentally Friendly Fabrication of Superhydrophobic Alkyl Ketene Dimer Coated MALDI Concentration Plates
Source: J Am Soc Mass Spectrom. 2017 Apr 12;28(8):1733–6. doi: 10.1007/s13361-017-1657-4 (PMC5507968; doi:10.1007/s13361-017-1657-4)

Online resource 2 – EMS_2

Journal of the American Society for Mass Spectrometry

"Simple and environmentally friendly fabrication of superhydrophobic alkyl ketene dimer coated MALDI concentration plates"

Joakim Romson, Johan Jacksén and Åsa Emmer*
*Corresponding author: aae@kth.se, KTH Royal Institute of Technology, School of Chemical Science and Engineering, Department of Chemistry, Analytical Chemistry, Stockholm, Sweden

EMS_2a. The printing substrates were made by partially melting a 10-µL pipette tip (polypropylene) over an open flame. Three different sizes are shown (left to right) largest to smallest. Grid is 5x5 mm.

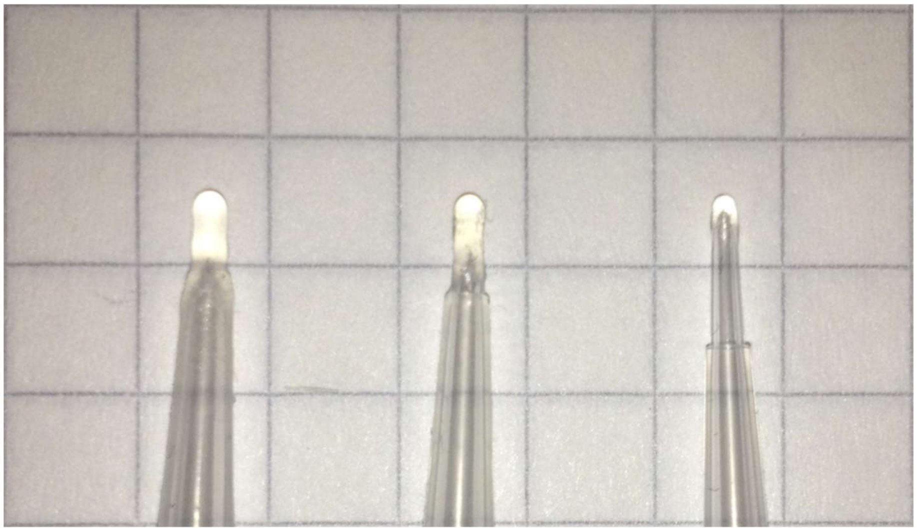

EMS_2b. Schematic of printing of the AKD plates.

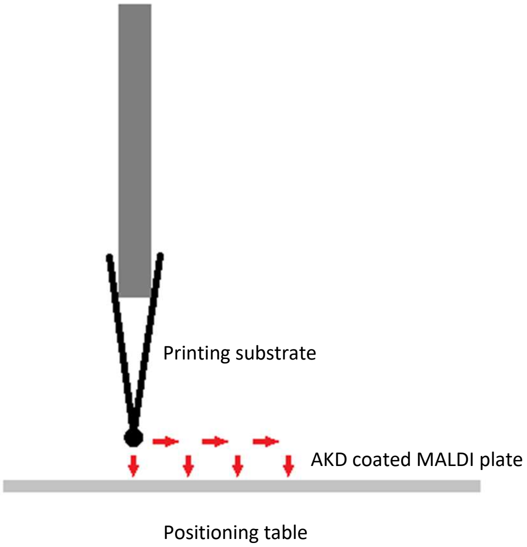

Supplement: Supplementary file 2 — (PDF 484 kb) [file 13361_2017_1657_MOESM2_ESM.pdf]
