# Supplementary material for: Simple and Environmentally Friendly Fabrication of Superhydrophobic Alkyl Ketene Dimer Coated MALDI Concentration Plates
Source: J Am Soc Mass Spectrom. 2017 Apr 12;28(8):1733–6. doi: 10.1007/s13361-017-1657-4 (PMC5507968; doi:10.1007/s13361-017-1657-4)

Online resource 3 – EMS_3

Journal of the American Society for Mass Spectrometry

"Simple and environmentally friendly fabrication of superhydrophobic alkyl ketene dimer coated MALDI concentration plates"

Joakim Romson, Johan Jacksén and Åsa Emmer*

*Corresponding author: aae@kth.se, KTH Royal Institute of Technology, School of Chemical Science and Engineering, Department of Chemistry, Analytical Chemistry, Stockholm, Sweden

EMS_3. Photographs of water droplets on the three MALDI plates. Top view from left to right: Sample sites without water droplets on GS (no concentration sites), AC (400 µm sites), and AKD (400 µm sites) plates. Bottom: 0.5 µl water deposited at each site.

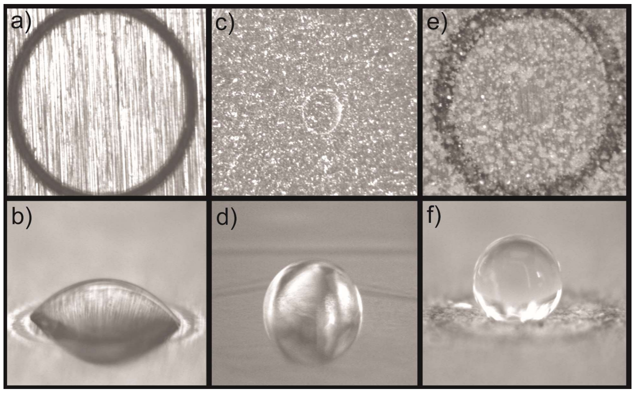

Supplement: Supplementary file 3 — (PDF 589 kb) [file 13361_2017_1657_MOESM3_ESM.pdf]
