# Supplementary material for: Simple and Environmentally Friendly Fabrication of Superhydrophobic Alkyl Ketene Dimer Coated MALDI Concentration Plates
Source: J Am Soc Mass Spectrom. 2017 Apr 12;28(8):1733–6. doi: 10.1007/s13361-017-1657-4 (PMC5507968; doi:10.1007/s13361-017-1657-4)
Supplement: Supplementary file 4 — (PDF 592 kb) [file 13361_2017_1657_MOESM4_ESM.pdf]

Online resource 4 – EMS_4

Journal of the American Society for Mass Spectrometry

"Simple and environmentally friendly fabrication of superhydrophobic alkyl ketene dimer coated MALDI concentration plates"

Joakim Romson, Johan Jacksén and Åsa Emmer*
*Corresponding author: aae@kth.se, KTH Royal Institute of Technology, School of Chemical Science and Engineering, Department of Chemistry, Analytical Chemistry, Stockholm, Sweden

EMS_4. The mean and the relative standard deviation of the obtained S/N for each peptide are presented for the three plates and two concentrations. 20 chosen positions on each spot blasted with 5*100 shots. (Manual = manual acquisition, Auot = automated acquisition; 100 random positions on each spot blasted with 100 shots).

The ratios of the mean S/N are shown to compare sensitivity for the four peptides, and an average of them presented as an overall comparison between the plates. P is the probability of falsely rejecting the null-hypothesis in the two-tailed t-test (t evaluated at 0.05 and 0.1), with the null-hypothesis that the mean S/N values do not differ significantly between the plates. The means that are not significantly different (P > 0.1) are marked in red, but are still included in the calculation of mean ratio. Normal distribution was assumed, an F-test was performed to check if variances differed significantly, no outliers were excluded.

**Manual**		**Peptide**	**AngII**	**AngI**	**GFpB**	**NT**	**Average ratio**	**SD**	**RSD**
**0.1 nM**	**GS S/N**	**Average**	206.2	28.50	45.10	219.8			
		**RSD**	0.3027	0.3255	0.2649	0.2511			
	**AC S/N**	**Average**	120.9	8.600	74.30	126.4			
		**RSD**	0.2665	0.5283	0.3073	0.4096			
	**AKD S/N**	**Average**	194.4	37.60	57.60	365.2			
		**RSD**	0.2137	0.1561	0.2814	0.1571			
	**AKD/GS**	**Ratio**	**0.9428**	**1.319**	**1.277**	**1.662**	**1.300**	0.2546	0.1958
		**P %**	> 10	< 5	> 10	< 5			
	**AKD/AC**	**Ratio**	**1.608**	**4.372**	**0.7752**	**2.889**	**2.411**	1.360	0.5641
		**P %**	< 5	< 5	> 10	< 5			
**0.05 nM**	**GS S/N**	**Average**	56.20	7.200	14.90	40.80			
		**RSD**	0.2492	0.2469	0.1885	0.2551			
	**AC S/N**	**Average**	38.80	4.900	14.30	37.90			
		**RSD**	0.4322	0.3920	0.3594	0.4394			
	**AKD S/N**	**Average**	59.90	14.10	18.60	116.9			
		**RSD**	0.2689	0.2700	0.2524	0.3532			
	**AKD/GS**	**Ratio**	**1.066**	**1.958**	**1.248**	**2.865**	**1.784**	0.7075	0.3966
		**P %**	> 10	< 5	< 5	< 5			
	**AKD/AC**	**Ratio**	**1.544**	**2.878**	**1.301**	**3.084**	**2.202**	0.7875	0.3576
		**P %**	< 5	< 5	< 5	< 5			
**Overall**	**AKD/GS**						**1.54**		
	**AKD/AC**						**2.31**		

Auto		Peptide	AngII	AngI	GFpB	NT	Average ratio	SD	RSD
**0.1 nM**	**GS S/N**	**Average**	26.90	1.900	5.000	16.90			
		**RSD**	0.5303	0.6842	0.4099	0.5335			
	**AC S/N**	**Average**	25.00	3.900	8.100	41.20			
		**RSD**	0.3432	0.5186	0.3424	0.5853			
	**AKD S/N**	**Average**	51.10	4.800	11.90	58.40			
		**RSD**	0.5213	0.2244	0.5240	0.2994			
	**AKD/GS**	**Ratio**	**1.900**	**2.526**	**2.380**	**3.456**	**2.565**	0.5638	0.2198
		**P %**	< 5	< 5	< 5	< 5			
	**AKD/AC**	**Ratio**	**2.044**	**1.231**	**1.469**	**1.417**	**1.540**	0.3040	0.1974
		**P %**	< 5	< 10	> 10	> 10			
**0.05 nM**	**GS S/N**	**Average**	13.90	1.000	3.100	4.500			
		**RSD**	0.3209	0.000	0.4194	0.6536			
	**AC S/N**	**Average**	6.600	1.100	2.000	6.800			
		**RSD**	0.5387	0.2727	0.5477	0.5948			
	**AKD S/N**	**Average**	20.00	1.300	5.300	13.90			
		**RSD**	0.2145	0.4925	0.1698	0.1490			
	**AKD/GS**	**Ratio**	**1.439**	**1.3**	**1.710**	**3.089**	**1.884**	0.7108	0.3773
		**P %**	< 5	> 10	< 5	< 5			
	**AKD/AC**	**Ratio**	**3.030**	**1.182**	**2.650**	**2.044**	**2.227**	0.6982	0.3136
		**P %**	< 5	> 10	< 5	< 5			
**Overall**	**AKD/GS**						**2.22**		
	**AKD/AC**						**1.88**		
