# Supplementary material for: Simple and Environmentally Friendly Fabrication of Superhydrophobic Alkyl Ketene Dimer Coated MALDI Concentration Plates
Source: J Am Soc Mass Spectrom. 2017 Apr 12;28(8):1733–6. doi: 10.1007/s13361-017-1657-4 (PMC5507968; doi:10.1007/s13361-017-1657-4)

Online resource 5 – EMS_5

Journal of the American Society for Mass Spectrometry

"Simple and environmentally friendly fabrication of superhydrophobic alkyl ketene dimer coated MALDI concentration plates"

Joakim Romson, Johan Jacksén and Åsa Emmer*

*Corresponding author: aae@kth.se, KTH Royal Institute of Technology, School of Chemical Science and Engineering, Department of Chemistry, Analytical Chemistry, Stockholm, Sweden

EMS_5. Spectra selected regarding highest S/N values obtained using manual acquisition (a-c), and using automated acquisition (d-f). 0.1 nM each of AngI, AngII, GFpB and NT. a and d) GS plate, b and e) AC plate, c and f) AKD plate.

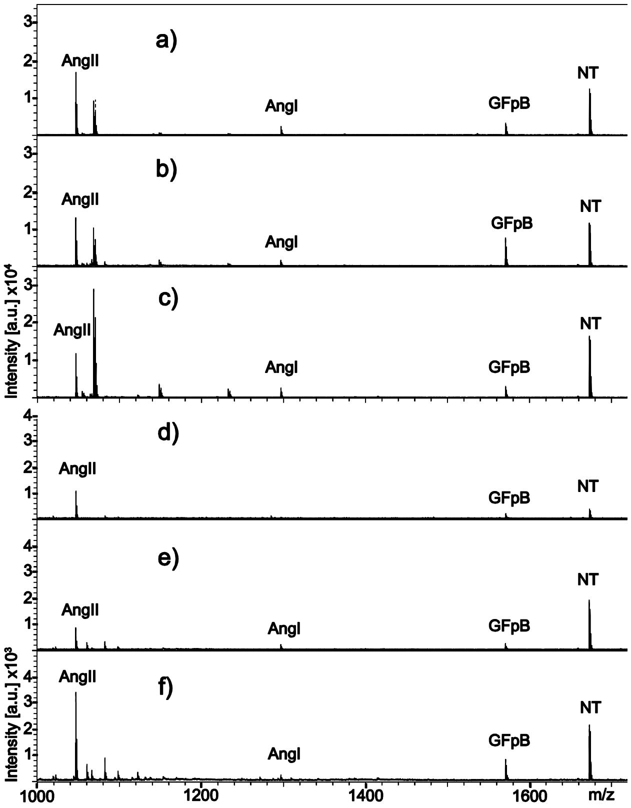

Supplement: Supplementary file 5 — (PDF 633 kb) [file 13361_2017_1657_MOESM5_ESM.pdf]
