# Supplementary material for: Simple and Environmentally Friendly Fabrication of Superhydrophobic Alkyl Ketene Dimer Coated MALDI Concentration Plates
Source: J Am Soc Mass Spectrom. 2017 Apr 12;28(8):1733–6. doi: 10.1007/s13361-017-1657-4 (PMC5507968; doi:10.1007/s13361-017-1657-4)
Supplement: Supplementary file 6 — (PDF 66 kb) [file 13361_2017_1657_MOESM6_ESM.pdf]

Online resource 6 – EMS_6

Journal of the American Society for Mass Spectrometry

"Simple and environmentally friendly fabrication of superhydrophobic alkyl ketene dimer coated MALDI concentration plates"

Joakim Romson, Johan Jacksén and Åsa Emmer*

*Corresponding author: aae@kth.se, KTH Royal Institute of Technology, School of Chemical Science and Engineering, Department of Chemistry, Analytical Chemistry, Stockholm, Sweden

EMS_6. Comparison of S/N value ratios for the four peptides obtained using the AKD plates in relation to AC using 5 mg/ml DHB in water as matrix. n=4. P is the probability of falsely rejecting the null hypothesis (that the mean S/N values do not differ significantly between the plates).

DHB		
**Ratio**	AKD/AC	P %
**AngII**	1.3	27
**AngI**	1.61	10
**GFpB**	1.25	40
**NT**	1.85	1.3
**Average ratio**	1.5	
**SD**	0.243	
**RSD**	16.2	
